# Implementation of machine learning in the clinic: challenges and lessons in prospective deployment from the System for High Intensity EvaLuation During Radiation Therapy (SHIELD-RT) randomized controlled study

**DOI:** 10.1186/s12859-022-04940-3

**Published:** 2022-09-30

**Authors:** Julian C. Hong, Neville C. W. Eclov, Sarah J. Stephens, Yvonne M. Mowery, Manisha Palta

**Affiliations:** 1 Department of Radiation Oncology, University of California, San Francisco, 1825 Fourth Street, Suite L1101, San Francisco, CA 94158 USA; 2 Bakar Computational Health Sciences Institute, University of California, San Francisco, San Francisco, CA USA; 3 Department of Radiation Oncology, Duke University, Durham, NC USA; 4 Department of Head and Neck Surgery and Communication Sciences, Duke University, Durham, NC USA; 5 Duke Cancer Institute, Duke University, Durham, NC USA

**Keywords:** Radiation therapy, Chemoradiation, Machine learning, Artificial intelligence, Quality improvement, Implementation

## Abstract

**Background:**

Artificial intelligence (AI) and machine learning (ML) have resulted in significant enthusiasm for their promise in healthcare. Despite this, prospective randomized controlled trials and successful clinical implementation remain limited. One clinical application of ML is mitigation of the increased risk for acute care during outpatient cancer therapy. We previously reported the results of the System for High Intensity EvaLuation During Radiation Therapy (SHIELD-RT) study (NCT04277650), which was a prospective, randomized quality improvement study demonstrating that ML based on electronic health record (EHR) data can direct supplemental clinical evaluations and reduce the rate of acute care during cancer radiotherapy with and without chemotherapy. The objective of this study is to report the workflow and operational challenges encountered during ML implementation on the SHIELD-RT study.

**Results:**

Data extraction and manual review steps in the workflow represented significant time commitments for implementation of clinical ML on a prospective, randomized study. Barriers include limited data availability through the standard clinical workflow and commercial products, the need to aggregate data from multiple sources, and logistical challenges from altering the standard clinical workflow to deliver adaptive care.

**Conclusions:**

The SHIELD-RT study was an early randomized controlled study which enabled assessment of barriers to clinical ML implementation, specifically those which leverage the EHR. These challenges build on a growing body of literature and may provide lessons for future healthcare ML adoption.

**Trial registration**: NCT04277650. Registered 20 February 2020. Retrospectively registered quality improvement study.

## Background

Artificial intelligence (AI) and machine learning (ML) have generated much enthusiasm in the healthcare space. Despite this, many obstacles remain to their adoption in routine clinical care. Among these are a lack of prospective data, need for trust from clinicians and patients, and logistical challenges in integration [1–5]. The need for this prospective deployment experience is critical, to verify accuracy and demonstrate usability and clinical value in the real world. As such, digital health innovations have had a limited clinical impact [6].

We previously completed one of the first randomized controlled studies of clinical ML, using an electronic health record (EHR)-based ML approach to identify patients at high risk for acute care (emergency department visit or hospitalization) during cancer radiation therapy (RT) [4]. These patients were then randomized to standard of care weekly evaluations (with ad hoc visits as deemed appropriate by the treating physician) versus mandatory twice-weekly evaluations. This study demonstrated that ML could appropriately identify high-risk patients and guide interventional strategies, reducing acute care rates in the high-risk population from 22.3% to 12.3%. Supportive management of patients with cancer is critical, with acute care resulting in detriments to patient outcomes, quality of life, treatment decisions, and costs, which have made it a priority to the Centers for Medicare and Medicaid Services [7–9].

The impact on clinical workflow is an important consideration to assess the hidden costs of clinical ML implementation [10]. This study focuses on describing the challenges encountered in the workflow of integrating a locally developed ML approach in a busy radiation oncology clinic during the course of the randomized controlled SHIELD-RT study.

## Results

### Deployment data extraction

One major identified barrier for the physics team was to develop a method for extracting data in real-time clinical practice. In aggregate, the below data extraction process required a median of 5 h (interquartile range [IQR] 4–5 h) per week of a medical physics resident’s time.

**Fig. 1:**
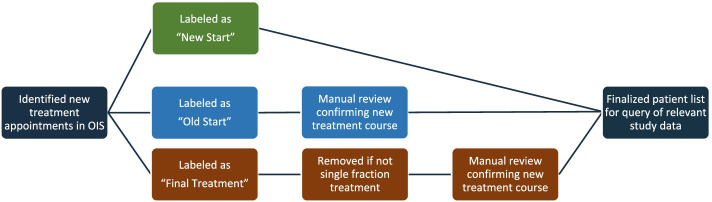
Patient identification workflow. New treatment courses were labeled as one of three potential options that required subsequent manual review

For the purposes of deployment, identification of new RT courses was required. One major challenge in practically identifying these courses was the labels used in the Aria oncology information system (OIS) (Varian Medical Systems, Palo Alto). During retrospective model development, this was simply queried to identify 8134 courses of radiotherapy completed from 2013 to 2016 [11]. In prospective development, identification of courses required queries through the scheduling system. The OIS designation at the time of SHIELD-RT designated new treatment appointments as three potential options: “new start” (new patient beginning new course), “old start” (patient with a prior OIS course starting new course) or “final treatment” (either final fraction of a multi-fraction treatment or start of a single fraction treatment) (Fig. 1). To identify courses during the first week of treatment, manual review was needed to verify “old starts” and for quality assurance to verify that single fraction treatments labeled as “final treatment” were indeed a new course of radiation therapy.

After identification of eligible treatment courses, RT data were extracted from the OIS, including details regarding the treatment course name, prescription, total dose, number of fractions, RT technique, and patient diagnosis based on International Classification of Diseases (ICD) codes.

Additional manual review was required to inspect draft (unsigned) prescriptions of sequential RT boosts and verify that they were an intended component of the treatment plan. This included subsequent radiation plans that were designed to deliver additional RT dose to a portion of the originally treated field within a single treatment course (e.g., a boost to a breast tumor bed following lumpectomy after primary whole breast treatment). Manual review of their inclusion was needed to accurately characterize a patient’s planned treatment course. Draft prescriptions typically represent planned treatment, but can also include boosts that are no longer intended (*e.g.,* due to radiation planning constraints). These draft prescriptions are sometimes pended unsigned at the start of treatment initiation and therefore not automatically aggregated.

### Machine learning deployment

Once patient RT data was identified, the process to generate ML predictions, randomize patients, and deploy clinical alerts was undertaken, requiring a median of 1.5 h per week (IQR 1–2 h) of the lead investigator’s time. From the OIS-generated patient list, the patient medical record number was used to query pre-treatment EHR data from the Duke enterprise data unified content explorer (DEDUCE) to provide additional input for the ML prediction [12]. DEDUCE aggregates data directly from the hospital and clinic operations via the Decision Support Repository (DSR), similarly to efforts utilizing data from institutional clinical data warehouses [13–15].

The combined OIS and EHR-queried data were then input into an aggregated R script to generate ML predictions. Patients identified as high risk (ML predicted 10% or greater risk of requiring acute care) were then entered into a REDCap database, which facilitated randomization, study documentation, and auditing [16]. Alerts were then manually placed in the OIS so that patients could be appropriately directed to supplemental visits, and the treating team was notified via manual emails. For auditing at a later time during the course of the study, the ML model was then run by two independent investigators and output verified.

### The clinical workflow

During treatment, alerts in the OIS prompted radiation therapists to direct high-risk patients who were randomized to the intervention arm to examination rooms for weekly mandatory supplemental visits. As previously reported, 79.7% (444 of 557) of mandatory supplemental evaluations were completed, with a median of 0 missed visits per course (IQR 0–1). Anecdotally, these were largely associated with missed alerts or patients forgetting about their supplemental evaluations especially in the context of variable scheduled times. These visits required an additional median of 5 min (IQR 5–10 min) of clinician time per visit [4].

## Conclusions

In this study, we identify specific challenges during the implementation of a randomized controlled study of EHR-based ML-directed clinical evaluations for cancer patients undergoing RT. We demonstrated specific barriers across the real-time data aggregation, ML deployment, and clinical workflow steps. While the challenges are specific to the radiation oncology domain, the broader barriers are important considerations for investigators and clinicians alike, as AI becomes increasingly relevant in the delivery of clinical care. These practical concerns are often not readily apparent or underestimated prior to clinical implementation, and can impact successful clinical use [1, 2, 10]. Streamlining the workflow to minimize deployment challenges is currently under discussion and investigation with institutional health ML oversight bodies as we work towards implementing our ML model into routine care.

One major obstacle was the need for real-time data aggregation, particularly in the context of data extraction from commercial products, such as our institutional OIS. Application programming interfaces (APIs) can improve integration with existing software. However, these opportunities do not consistently exist, presenting a barrier to institutionally developed and commercial solutions alike. Furthermore, we demonstrated that as the data were not stored in a fashion conducive for this use case, additional, in some cases manual, evaluation may be needed to obtain the required information. Modifications of OIS course start naming conventions and consistent entry of draft prescriptions may improve automation and reduce the need for manual review.

Disparate information systems represent a second challenge. Cancer care, including RT, frequently involves multiple information systems that capture data salient to clinically relevant decisions. This includes the EHR and OIS, as well as other sources (pathology information systems), procedure data, and genomic data. Some of these elements are aggregated in the EHR, but typically in an unstructured format that makes real-time utilization challenging. The planned integration of data derived from clinical free-text will further introduce challenges in real-time data integration [17]. Our team is currently working towards a unified, rather than ad hoc data stream to improve linkage and clinical deployment.

Finally, we developed a clinical workflow that minimizes the number of touch points during the clinic day, integrating a direct OIS alert to the radiation therapy team at a treatment machine and the clinician responsible for the supplemental visits. Rates of supplemental visit completion were high, and overall clinician time was efficient.

This study does have limitations, including a specific use case and single institution. These may limit the generalizable lessons from our implementation, though this study demonstrates broader themes in ML implementation. This algorithm was also deployed during a 6-month period. Routine clinical deployment or longer-term prospective studies require more prolonged implementation periods, which introduce the risk of other confounders, such as automation bias or distributional shift, requiring regular quality assurance [18].

This early randomized study of ML-directed care demonstrates the potential for ML to guide systematic, clinically meaningful differences at the point of care. However, many challenges arose that required staff time and effort, and these must be streamlined for clinical deployment and routine adoption.

## Methods

### Ethics, consent, and permissions

SHIELD-RT was a prospective, randomized controlled quality improvement (QI) study, which was approved by the Duke University Medical Center Institutional Review Board (Pro00100647) and registered on ClinicalTrials.gov (NCT04277650). As a QI study, study consent was not required.

### SHIELD-RT study details

The methods of the SHIELD-RT study have been previously described [4]. This study included all adult outpatient RT courses with or without concurrent systemic therapy from January 7, 2019 to June 30, 2019 at the Duke Cancer Institute. Total body irradiation courses were excluded due to planned admissions.

The ML model was previously developed, and source code is available online [11]. This was deployed and run weekly to identify high-risk patients who had started RT in the current week, with > 10% predicted risk of requiring acute care in the form of an emergency department (ED) visit or hospital admission. High-risk patients were subsequently randomized to standard of care, which consists of weekly on-treatment evaluations by the treating radiation oncologist, or the addition of a mandatory second weekly evaluation, typically performed by a clinician on the primary treating team (attending physician, resident physician, advanced practice provider, or nurse clinician). Both arms allowed for additional evaluations as indicated by the treating physician. The primary endpoint of this study was the rate of acute care visits during courses of RT, with secondary endpoints including the rate of acute care visits during RT and the 15 days following treatment, rates of missed supplemental evaluations, and reasons for acute care (grouped by those designated as potentially preventable by CMS [9]).

### Implementation data collection

During the course of the study, investigators at each stage of implementation logged their time spent on the various tasks needed for deployment. Clinician time was also documented in formal EHR clinical visit notes. Each team also described their workflows to facilitate future reproduction for routine clinical implementation.

## Data Availability

Data sharing is not applicable to this article as no datasets were generated or analyzed during the current study.

## Abbreviations

AI: Artificial intelligence
API: Application programming interfaces
CMS: Centers for Medicare and Medicaid Services
DCR: Decision support repository
DEDUCE: Duke enterprise data unified content explorer
ED: Emergency department
EHR: Electronic health record
ICD: International classification of diseases
IQR: Interquartile range
ML: Machine learning
OIS: Oncology information system
QI: Quality improvement
RT: Radiation therapy
SHIELD-RT: System for High Intensity EvaLuation During Radiation Therapy
